# Novel Biogenic Aggregation of Moss Gemmae on a Disappearing African Glacier

**DOI:** 10.1371/journal.pone.0112510

**Published:** 2014-11-17

**Authors:** Jun Uetake, Sota Tanaka, Kosuke Hara, Yukiko Tanabe, Denis Samyn, Hideaki Motoyama, Satoshi Imura, Shiro Kohshima

**Affiliations:** 1 Transdisciplinary Research Integration Center, Minato-ku, Tokyo, Japan; 2 National Institute of Polar Research, Tachikawa, Tokyo, Japan; 3 Faculty of Science, Chiba University, Chiba, Chiba, Japan; 4 Graduate School of Science, Kyoto University, Kyoto, Japan; 5 Institute for Advanced Study, Waseda University, Shinjuku-ku, Tokyo, Japan; 6 Department of Mechanical Engineering, Nagaoka University of Technology, Nagaoka, Nigata, Japan; 7 Wildlife Research Center, Kyoto University, Kyoto, Kyoto, Japan; University of California Davis, United States of America

## Abstract

Tropical regions are not well represented in glacier biology, yet many tropical glaciers are under threat of disappearance due to climate change. Here we report a novel biogenic aggregation at the terminus of a glacier in the Rwenzori Mountains, Uganda. The material was formed by uniseriate protonemal moss gemmae and protonema. Molecular analysis of five genetic markers determined the taxon as *Ceratodon purpureus*, a cosmopolitan species that is widespread in tropical to polar region. Given optimal growing temperatures of isolate is 20–30°C, the cold glacier surface might seem unsuitable for this species. However, the cluster of protonema growth reached approximately 10°C in daytime, suggesting that diurnal increase in temperature may contribute to the moss’s ability to inhabit the glacier surface. The aggregation is also a habitat for microorganisms, and the disappearance of this glacier will lead to the loss of this unique ecosystem.

## Introduction

Many psychrophilic and psychrotolerant microorganisms inhabit supraglacial environments, which have recently been recognized as an important biome [1]. Cryonite granules, dark spherical aggregates typically 1 mm in diameter have been frequently observed on ablation zones of glaciers in many parts of the world [2]. These cryoconites consist of mineral particles, organic matter, and microorganisms, which are mainly formed by the aggregation of filamentous cyanobacteria [3]. These cryoconite harbor a diverse range of microorganisms, and studies of their molecular diversity have revealed microbial communities of bacteria [4] and archaea [5], [6], as well as algae, fungi, amoebas, and invertebrates such as tardigrades [5]. These microbial communities play an important role in the carbon and nitrogen cycles on the glacier [1], [7].

Other types of biological aggregations have been reported from supraglacier ecosystems in Iceland and Alaska, namely, globular moss aggregations known as ‘glacier mice’ [8], [9] or ‘moss polster’ [10]. These are lenticular moss cushions (0.02 to 0.1 m in diameter) and are composed of a moss envelope covering an internal clast formed from glacial sediment and airborne particles [11]. These moss cushions are expected to impact the ecology and nutrient cycle of the supraglacial ecosystem [11], and also provide a favorable habitat for a variety of invertebrates, including Collembola, Tardigrades, and Nematoda [12].

Previous biological studies have frequently examined mid-latitude and polar glaciers, however, the tropical glaciers are have been studied rarely, except for New Guinea [13]. In equatorial Africa, glaciers persist in three major mountain regions (Mt. Kilimanjaro in Tanzania, Mt. Kenya in Kenya, and the Rwenzori Mountains in Uganda), which have not been previously been targeted in surveys of glacier biology. The Rwenzori glaciers are shrinking rapidly and are expected to disappear by 2020 due to climatic warming [14] and/or lowered humidity and lowered cloudiness [15], as measured by aerial photography and satellite imagery [14].

During a biological field survey on a glacier near the summit of Mt. Stanley, the highest peak in Uganda and in the Rwenzoris, we found a large, black bioaggregation (average long and short axes: 18.1 mm and 12.7 mm) in the supraglacial environment, greater than cryoconites. Examination revealed that these granular structures were formed by filamentous moss gemmae and protonema, not cyanobacteria. This is the first report to describe this habitat for such a structure, which we classified as a “glacial moss gemmae aggregation” (GMGA). In order to identify the material we measured the structure (size and mass) and isolated the dominant moss species using both culture and molecular techniques. Furthermore, we examined the photosynthetic activity of isolates under various temperature and radiation conditions.

## Materials and Methods

### Glacier characteristics and sampling

**Figure 1:**
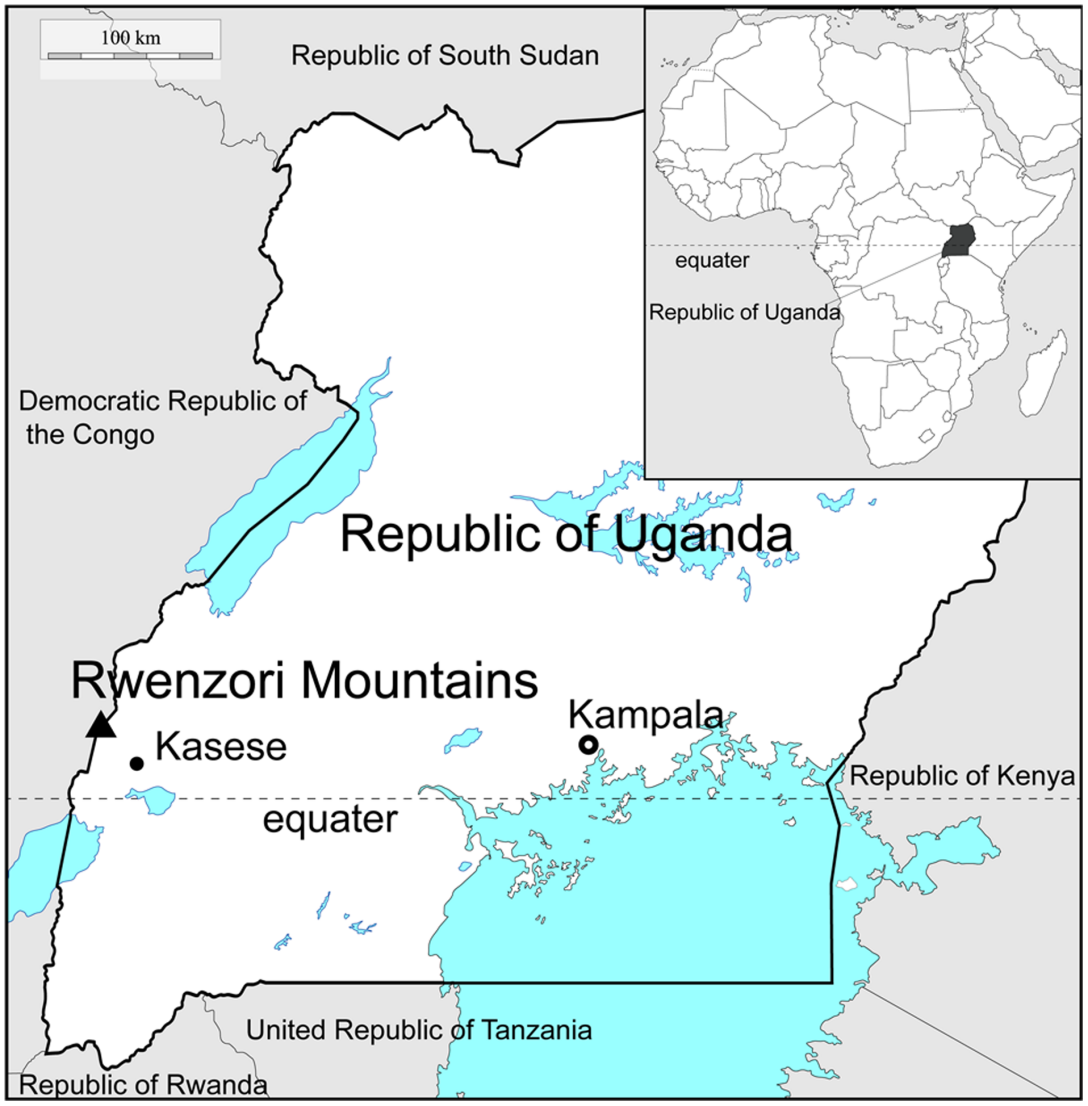
Location map of Rwenzori Mountains in Republic of Uganda.

**Figure 2:**
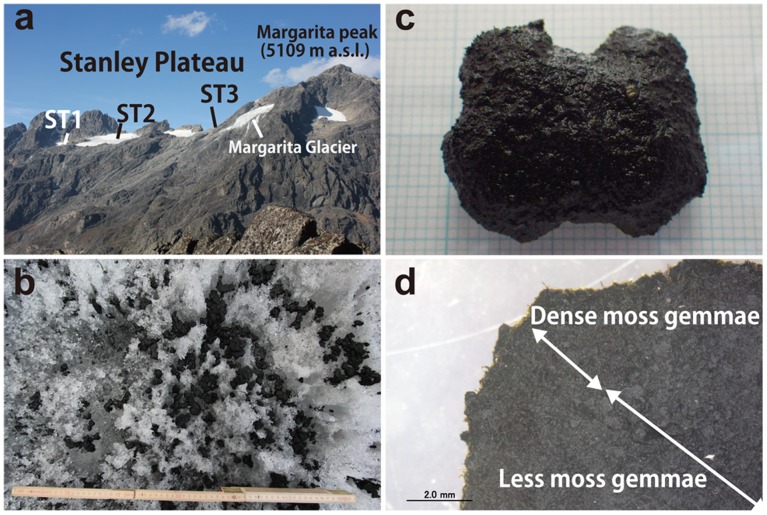
Research site and glacier moss gemmae aggregation (GMGA). a) Stanley Plateau glacier and Margarita Peak from Mt. Baker, b) Glacier ice surface covered by GMGAs, c) GMGAs (grid cells beneath GMGA are 1×1 mm), d) Cross section of GMGA (scale bar: 2 mm).

The Rwenzori Mountains (5109 m above sea level) contain the third-highest mountain in Africa, and are straddling the equator along the border of Uganda and the Democratic Republic of the Congo (Fig. 1). Since the LIA (Lac Gris Stage: 19^th^ centry or just before), glaciers in the Rwenzoris have been shrinking; in 1906 the Elena glacier was estimated to cover 6.5 km^2^ [16] and by 2003 it had decreased to approximately 1 km^2^ [14]. During this period, glaciers on Mts. Emin, Gessi, and Luigi disappeared completely, leaving only glaciers remaining on three major peaks: Mts. Speke, Baker, and Stanley. In this study, we surveyed Stanley Plateau, the largest glacier on Mt. Rwenzori. Stanley Plateau is a flat sloped glacier that flows from Mt. Stanley’s Alexandra Peak, and is around 1 km long and 0.1–0.3 km wide (Fig. 2a).

In February 2012 and 2013, we collected surface ice samples, including biological debris, at three sites: ST1 (N00°22′31.3″, E29°52′40.26″), ST2 (N00°22′34.74″, E29°52′37.2″) and ST3 (N00°22′52.32″, E29°52′24.6″). At each site, 5 samples were collected from different 0.1×0.1 m areas and stored in 50 ml plastic bottles for cell counts, and 5 samples from different areas (samples size not measured) and placed in 8 ml plastic bottles for DNA analysis and isolation. In the glacier foreland, located about 10 m from ST1, we collected shoots of bryophyte on dried GMGAs and placed them in 8 ml plastic bottles with RNAlater (Life Technologies, Carlsbad, USA). All samples were collected using pre-cleaned stainless steel scoops and spoons.

Samples for cell counts were fixed with 3% formaldehyde and stored at room temperature. All other samples were kept cold around 0°C in large stainless steel vacuum flasks with glacial ice samples until transport to Kasese, Uganda, the closest city to Rwenzori Mountains National Park. There, samples for molecular analysis were kept frozen around −20°C and samples for isolation were kept cold around 0°C, until they could be transported to the lab for analysis at the National Institute of Polar Research (Tokyo, Japan).

In the field, the internal temperature of the GMGAs at ST1 was measured using a waterproof temperature logger (R-52i; T&D, Matsumoto, Japan between 9–13 Feb. 2013) with 0.6 m sensor probe (TR-5106; T&D, Matsumoto, Japan). Probes were inserted into the center of two GMGAs and were monitored by camera (Optio WG-2: Ricoh, Tokyo, Japan) at intervals to ensure that the measuring apparatus was not disturbed.

### Microscopic observation of biological materials on ice surface

The samples were cold-preserved prior to isolation and identification of species. After formaldehyde fixation, 0.1–0.4 ml of 12–60 fold diluted samples were filtered through a hydrophilic polytetrafluoroethylene membrane (Omnipore JGWP01300; Merck Millipore, Billerica, USA) with diameter 13 mm and pore size 0.2 µm. We observed and counted cell concentrations from one-quarter of the membrane, using a fluorescent microscope (IX71 and 81; Olympus, Tokyo, Japan).

### 18S r RNA gene molecular cloning

DNA of approximately 0.3 g was extracted from samples ST1, ST2, and ST3 using the Fast DNA SPIN Kit for soil (MP Biomedicals, Santa Ana, USA) according to the manufacturer’s instructions. Extracted DNA was diluted to 1.62 ng/µl with water (Ambion Nuclease-Free Water; Life Technologies, Carlsbad, USA). Five aliquots from each site were combined for DNA amplification. Thermal cycling was performed with an initial denaturation step at 98°C for 3 min, followed by 25 cycles of denaturation at 98°C for 10 s, annealing at 55°C for 30 s, and elongation at 72°C for 1.5 min, using Ex Taq HS DNA polymerase (Takara, Shiga, Japan) and the primer pair of Euk A (5′-ACCTGGTTGATCCTGCCAGT-3′) and EukB (5′-GATCCTTCTGCAGGTTCACCTAC′). Cycling was completed by a final elongation step at 72°C for 3 min. The PCR-amplified DNA fragments were cloned into the pCR4 vector of the TOPO TA cloning kit (Invitrogen, Carlsbad, USA). Clones obtained from the libraries were sequenced using the 3130×l Genetic Analyzer (Life technologies, Carlsbad, USA) at the National Institute of Polar Research. All sequences were assembled using CodonCode Aligner (CodonCode Corporation, Centerville, USA) and assembled full-length sequences of 18S rRNA were aligned with the eukaryotic Silva database [17] using mothur ver. 1.27.0 [18]. Tentative chimeric sequences were removed using both the reference and *de novo* modes of Uchime [19] implemented in mothur software package. All good-quality sequences with more than 97% similarity were clustered into operational taxonomic units (OTU).

### Isolation of moss and molecular identification

Fragments of cold-preserved GMGA samples were inoculated in liquid Bold’s basal medium (BBM) [20] in a laminar flow bench and incubated at 4°C for 1 month. Protonemata that grew directly from observed gemmae (Fig. 2 c,d) were transplanted to fresh BBM liquid medium and a 1-month-incubation was repeated. Isolated protonemata were kept in BBM and 1.5% agar medium before extraction and analysis. DNA of a single cluster of protonema in liquid medium was extracted with the Fast DNA SPIN Kit for soil, and 4 different regions (18S rRNA; chloroplast genes, *trn*L, *rps*4 and *atpB*-*rbc*L intergenic spacer; and mitochondria gene, *nad*5) were amplified by using Ex Taq HS DNA polymerase (Takara, Shiga, Japan). Thermal cycling for 18S rRNA was carried out following Remias *et al.* [21], with 35 cycles of denaturation at 98°C for 10 s, annealing at 54°C for 30 s, and elongation at 72°C for 1 min 45 s, using the primer pair NS1 (5′-GTAGTCATATGCTTGTCTC-3′) and 18L(5′-CACCTACGGAAACCTTGTTACGACTT-3′). For chloroplast *trn*L, thermal cycling was performed with 35 cycles of denaturation at 98°C for 10 s, annealing at 60°C for 30 s, and elongation at 72°C for 1 min 45 s and primer pair trnC (5′- CGAAATCGGTAGACGCTACG-3′) and trnF (5′-ATTTGAACTGGTGACACGAG-3′), following Taberlet *et al.* [22]. For chloroplast *rps*4, thermal cycling was performed according to Nadot *et al.* [23] and Souza-Chies *et al.* [24], with 35 cycles of denaturation at 98°C for 10 s, annealing at 55°C for 30 s, and elongation at 72°C for 1 min 45 s using primer pair rps5 (5′-ATGTCCCGTTATCGAGGACCT-3′) and trnS (5′-TACCGAGGGTTCGAATC-3′). For chloroplast *atpB*-*rbc*L intergenic spacer, thermal cycling was performed according to Chiang *et al.* [25], with 35 cycles of denaturation at 98°C for 10 s, annealing at 49°C for 30 s, and elongation at 72°C for 30 s using primer pair *atpB*-1 (5′-ACATCKARTACKGGACC-3′) and *rbc*L-1 (5′-AACACCAGCTTTRAATCCAA-3′). Lastly, for mitochondria nad5, thermal cycling was performed according to Shaw *et al.* [26], with 35 cycles of denaturation at 98°C for 10 s, annealing at 52°C for 30 s, and elongation at 72°C for 1 min 45 s using primer set *nad*5F4 (5′-GAAGGAGTAGGTCTCGCTTCA-3′) and nad5R3 (5′-AAAACGCCTGCTGTTACCAT-3′). Some of shoots of Bryophyta on dried GMGAs were picked up by tweezers and DNA was analyzed by same method as isolated protonema.

### Photosynthetic rate of GMGA and isolate

Photosynthetic rate at 7 different incubation temperatures (5, 10, 15, 20, 25, 30, and 40°C) was measured using a pulse amplitude modulation (PAM) fluorometer (Water-PAM, Waltz, Effeltrich, Germany) with Win-control software for control and analysis following Tanabe *et al.* [27]. PAM fluorometer is useful to measure the electron transport rate (ETR) of isolate underdifferent incubation factor [27]. For incubation temperatures of 5, 10, and 15°C, photosynthetically active radiation (PAR) intensities were 3, 64, 94, 144, 215, 305, 422, 687, and 1000 mmol photons/m^2^/s. After these measurments, we had changed to another PAM device, because this device is obviously unstable only under 40°C due to mechanical trouble. Then, we used another device and measured again from 20°C. Results of 20, 25, 30°C are almost same as previous analysis, and measurement was stable at 40°C in next time. For incubation temperatures of 20, 25, 30, and 40°C, PAR intensities were 8, 62, 92, 140, 209, 297, 412, 674, and 986 mmol photons/m^2^/s. After a 30 s exposure, a saturating pulse of >2000 mmol photons/m^2^/s was applied for 0.4 sat 5°C in a temperature-controlled incubator. The gain value of the photoelectric multiplier (PM-Gain) was set to 3 for all measurements. After incubation of each sample at 5°C for 60 min in dark conditions, a tissue sample of GMGA and isolated protonemata were transferred to the quartz cuvette of the fluorometer. After measurement, incubation temperature was raised by 5°C to 10°C and incubated for 1 h, after which the temperature was raised by 5°C again and incubated for 1 h repeatedly until incubation temperature was 40°C. Light curves were obtained by running a rapid light curve protocol in Win-control software. The photosynthetic rate expressed as relative electron transport rate *rETR* [28] was as follows:
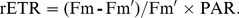
(1)


Here, F and Fm’ are the transient and maximum fluorescence levels at certain actinic light intensities at a given time and (Fm’–F)/Fm’ indicates Photosystem II (PSII) yield. Non-photochemical quenching (NPQ) was calculating by the following equation:
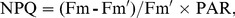
(2)where Fm is the maximum fluorescence level of non-illuminated samples.

### Ethics Statement

Uganda Wildlife Authority and Uganda National Council for Science and Technology authorized all field researchs in Rwenzori Mountains National Park.

## Results

### Morphological features of GMGA

**Figure 3:**
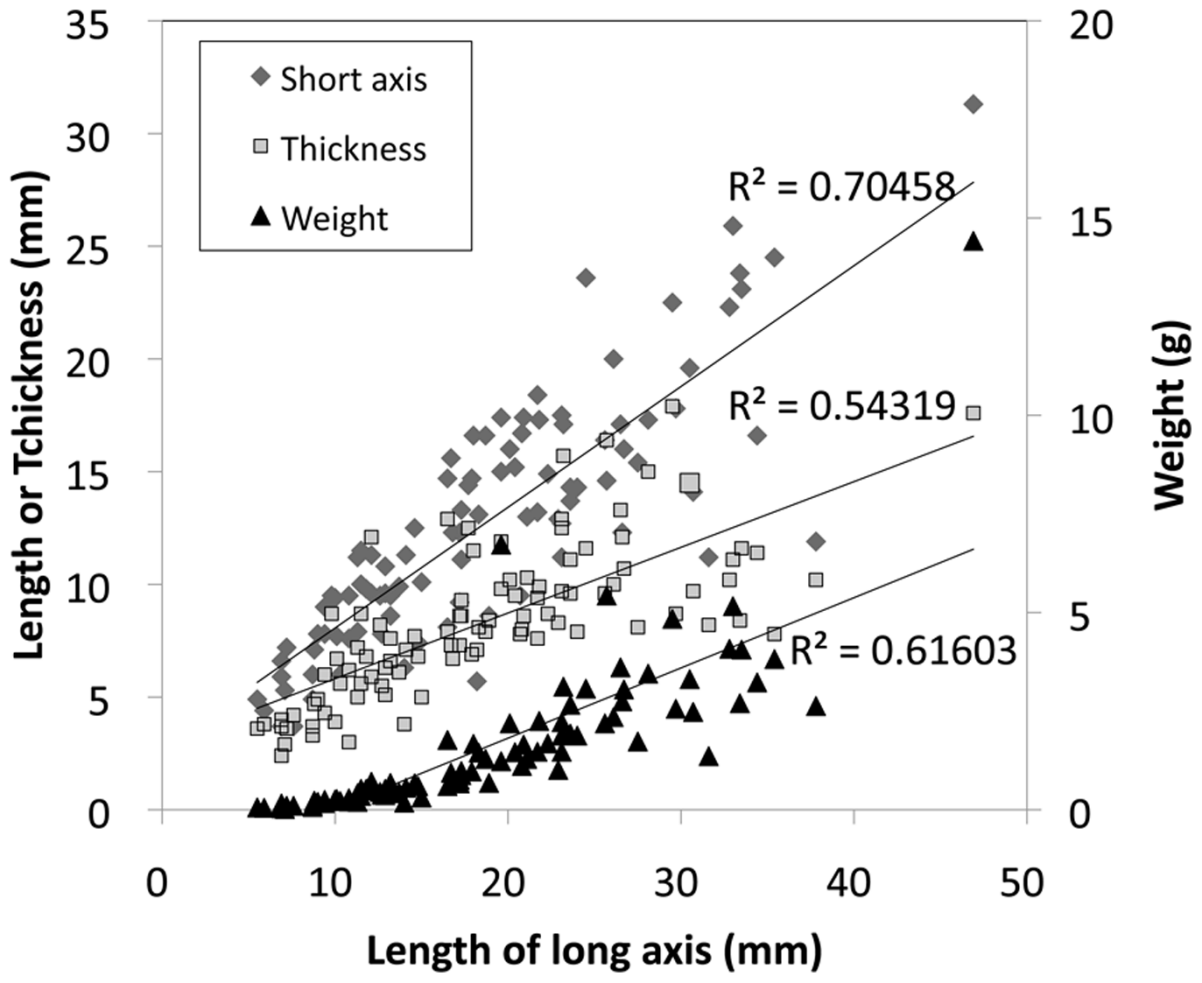
Size (long and short axes, thickness) and mass distribution of GMGAs.

**Figure 4:**
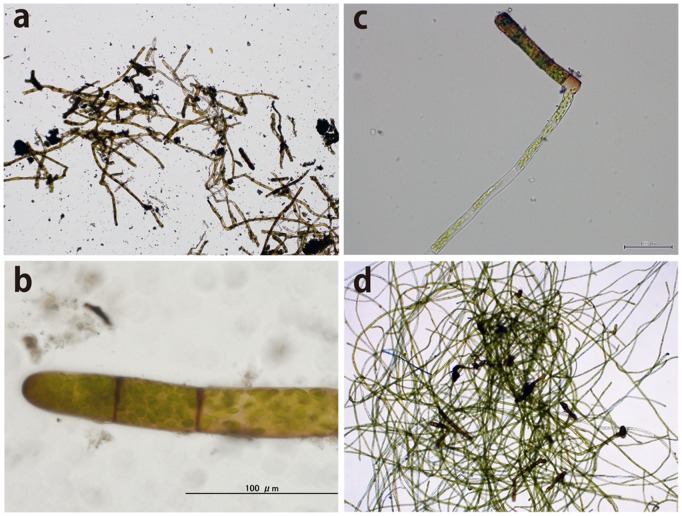
Cells of moss gemmae and protonemata. a,b) Moss protonemal cells formed the main frame of the GMGA (scale bar: 100 µm), c) Moss protonema grew from gemmae below 4°C (scale bar: 100 µm), d) protonemal cells for molecular identification after incubation below 4°C for 1 month in liquid Bold’s basal medium.

We found ellipsoidal blackish bioaggregations covering the glacier surface at ST1 (Fig. 2b). We collected and sampled 96 of these bioaggregations, as well as measured their long and short axes, thickness, and mass (Fig. 2c,d). The average long axis, short axis, thickness, and mass were 18.7 mm, 12.7 mm, 8.3 mm, and 1.6 g, respectively (Fig. 3), and these aggregations were clearly larger than cryoconites (average diameter: 1.1 mm; 3). Short axis length was well correlated with long axis length (R^2^ = 0.705), but not as well correlated with thickness and mass (R^2^ = 0.543 and 0.616, respectively). This means that the structure of this bioaggregation is not spherical but is instead flattened. The bioaggregation was composed of many gemmae and protonema. The gemmae were germinating and developing filamentous protonema, and gemmae were formed repeatedly on protonema. Many moss gemmae were observed especially on the surface of the bioaggregation (Fig. 4a,b), from the top 1–2 mm of the cross section (Fig. 2d). The main framework of these structures was formed by moss gemmae, so we named this structure as “glacial moss gemmae aggregation (GMGA)”.

The gemmae are filamentous, composed of 1–2 rows of 2–20 cells with slightly thickened brownish cell walls, 100–200 um long in maximum. These morpholocial characteristics were well agree with rhizoidal gemmae of cosmopolitan moss, *Ceratodon purpureus* (Hedw.) Brid. described by Imura and Kanda (1986) based on Antarctic specimens [29].

### Molecular identification of moss species

We obtained a total of 81 clones from the GMGAs by 18S rRNA gene PCR-cloning. Sixty-three clones (77.8%) were clustered into the same OTU (AB858433: Table 1). The remaining 18 clones were of cercozoa, green algae, and fungi.

**Table 1:** Sequences list of five different genetic regions from three different sample types (1: GMGA_cloning, 2: isolate protonema and 3: dried GMGA_cloning) and their closest relatives.

Gene type	Geneticregion	Sample type	Accessionnumber	Length(bp)	Closestrelative specie	Accession numberof relative	Identity(%)	sequencematch (bp)
RibosamalRNA gene	18S rRNA	1: GMGA_cloning	AB858433	1819	*Ceratodon* sp.AM2008N12	KC291530	99.8	1721/1724
					*Ceratodon purpureus*	Y08989	99.7	1751/1757
		2: isolate protonema			*Ceratodon purpureus*	KC291530	99.9	1677/1678
					*Ceratodon purpureus*	Y08989	99.7	1673/1679
					GMGA_cloning(this study)	AB858433	100	1679/1679
		3: driedGMGA_cloning	AB872997	1697	*Bryum caespiticium*	AF023703	100	1697/1697
Chloroplastgene	*rps*4	2: isolateprotonema	AB848717	674	*Ceratodon purpureus*	FJ572605	100	623/623
					*Ceratodon purpureus*	FJ572589	100	625/625
					*Ceratodon purpureus*	AF435271	100	561/561
					*Ceratodon purpureus*	AY908122	100	652/652
					*Trichodon cylindricus* *	AY908125	94.1	622/661
		3: driedGMGA_cloning	AB872999	684	*Bryum cyathiphyllum*	AF521683	99.9	667/668
	*trn*L	2: isolate protonema	AB848718	482	*Ceratodon purpureus*	FJ572485	100	482/482
					*Ceratodon purpureus*	AF435310	100	482/482
					*Glyphomitrium* *humillimum* *	EU246911	94.2	438/465
		3: driedGMGA_cloning	AB873000	520	*Bryum cyathiphyllum*	AY150351	100	492/492
	*atp*B-*rbc*Lintergenic spacer	2: isolate protonema	AB980065	637	*Ceratodon purpureus*	AY881031	100	621/621
					*Ceratodon purpureus*	AY881034	100	621/621
					*Ceratodon purpureus*	AY881052	100	598/598
					*Cheilothela chloropus* *	AY881063	89.9	571/635
Mitocondorialgene	*nad*5	2: isolateprotonema	AB848719	1112	*Ceratodon purpureus*	AY908859	99.9	1093/1094
					*Ceratodon purpureus*	AY908862	99.9	1090/1091
					*Trichodon cylindricus* *	AY908863	99.5	1089/1095
		3: driedGMGA_cloning	AB872998	1107	*Bryum argenteum*	AY908945	100	1082/1082

*show second highly related species.

The 18S rRNA, *rps*4, *trn*L, *atp*B-*rbc*L intergenic spacer and *nad*5 gene sequence of the isolated protonemata (Fig. 4c,d) that grew from the observed gemmae and shoots on dried GMGA were summarized in Table 1. These high-percentage matches (more than 99.9% similarity) from five different regions of the protonemata show that the isolated moss is indeed *C*. *purpureus*. Also results from four different regions of shoots on dried GMGA show that this specie belonging to genus: *Bryum*, however we could not identify species level from these regions.

### Optimum temperature and PAR of GMGA and isolated protonemata of *C. purpureus*

**Figure 5:**
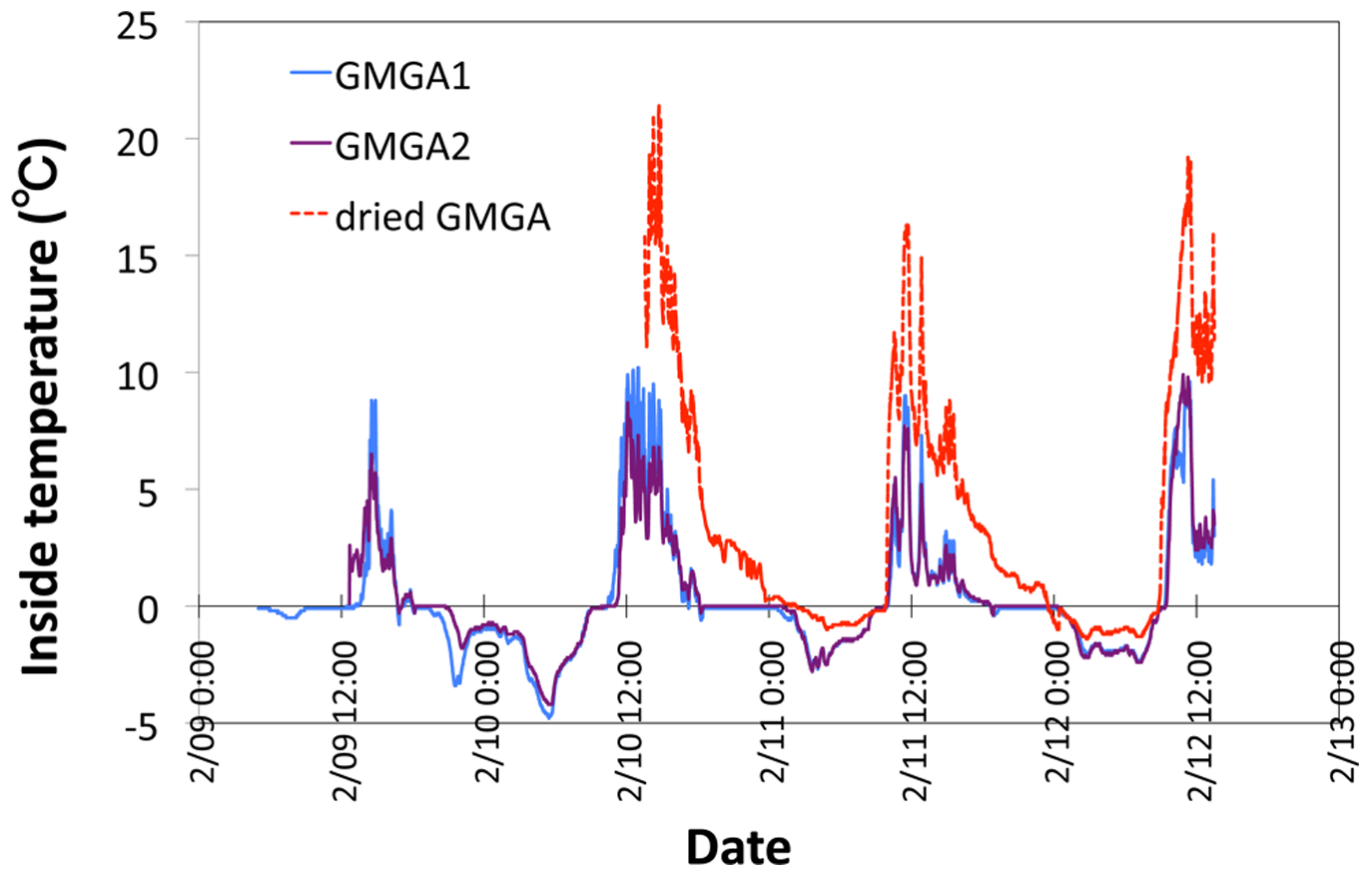
Internal changes in temperature of 2 GMGA and 1 dried GMGA left on a rock during the 2013 research period (February 9–12, 2013).

Internal (center) temperature changes was measured during the 2013 field season of two *in situ* GMGAs in ST1 and one dried GMGA found on a rock in glacier foreland (Fig. 5). Temperature change data show clear diurnal cycles with daily exposure to below-freezing temperatures daily. Maximum temperatures reached 8–10°C for *in situ* GMGAs and above 20°C for dried GMGA. The daytime increase in temperature was due to absorption of thermal radiation, but was variable due to decreases in radiation from frequent cloud cover and cooling by glacier ice.

**Figure 6:**
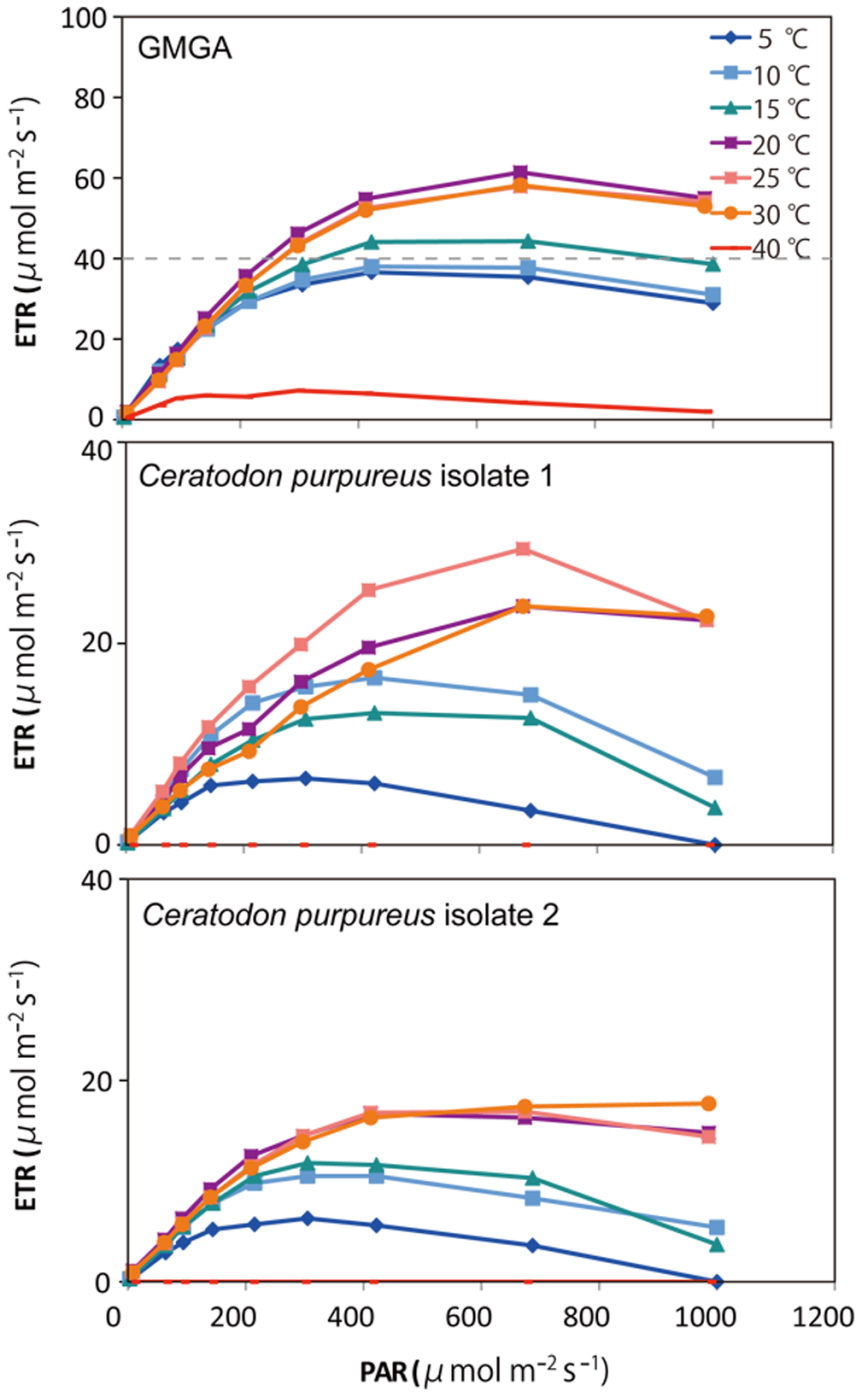
Photosynthetic light curves of GMGA and the two isolates under different incubation temperatures (from 5°C to 40°C) using a pulse amplitude modulation fluorometer.

A photosynthetic light curve was measured using a PAM fluorometer under different temperatures of GMGA and the two isolates (Fig. 6). The ETR of GMGA and isolates is high between 20–30°C, and highest at 25°C for GMGA. Electron transport was detected in all three samples even at temperatures as low as 5°C, but ETR was zero or extremely low at 40°C. These results indicate that the optimum temperatures of GMGA and isolated *C. purpureus* are around 25°C. The ETR of GMGA and the isolates was high at low PAR levels (305 and 422 µmol/m^2^/s) at 5–15°C; however, ETR was high at a medium PAR level (687 µmol/m^2^/s) at 20–30°C (Fig. 5). Therefore, the optimal PAR value for *in situ* temperatures (0–10°C) is likely between 305–422 µmol/m^2^/s. The highest ETR value in GMGAs was 674 µmol/m^2^/s, which was approximately twice that of the other two isolates (Fig. 6).

### Distribution of GMGAs on the glacier

**Figure 7:**
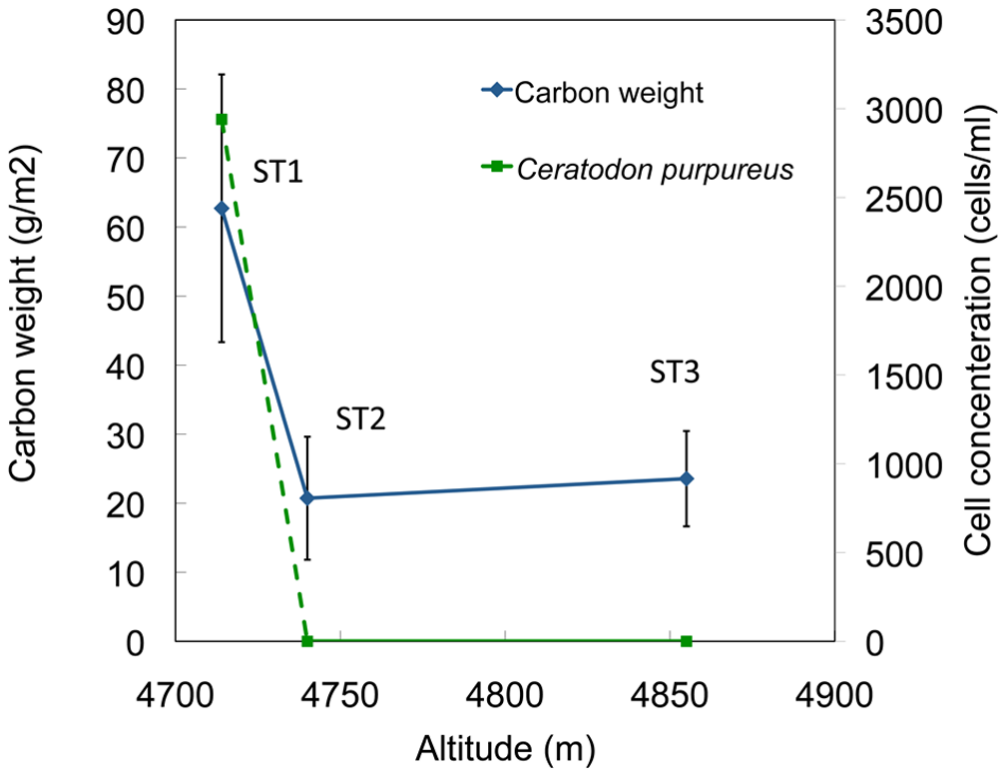
Distribution of carbon mass and *Ceratodon purpureus* cell concentrations on Stanley Plateau Glacier.

GMGAs of *Ceratodon purpureus* were observed only at site ST1, the glacier terminus (Fig. 7). The organic carbon mass (62.72±19.39 g/m^2^) was highest at the terminus. This record is higher than current highest glacial organic carbon mass (38.5±12.4 g/m^2^) from Qiyi Glacier, China [30]. Moreover, organic carbon mass at our sites without observed GMGAs (ST2∶20.73±8.91 g/m^2^, ST3∶23.55±6.89 g/m^2^) were roughly equal to the average high organic carbon mass at Qiyi Glacier (mean: 25.4±16.5 g/m^2^) [26].

## Discussion

### Glacial Moss Gemmae Aggregation (GMGA) is a novel moss aggregation

Mosses, in the form of “glacier mice”, have been previously recorded from supraglacial habitats [11], [12]; however, the structure of GMGA is completely different from that of “glacier mice”. Whereas “glacier mice” are formed by the moss shoots, that level of cellular differentiation was not detected in GMGAs. These findings report first description of developing moss gemmae and protonema in the supraglacial environments. *Ceratodon purpureus*, which formed the GMGA observed in this study, is a cosmopolitan moss species widely distributed throughout entire continents [31] and is known to grow in extreme environments (i.e. polluted sites including highway shoulders and on coal and heavy metal mine tailings) [32]. *Ceratodon purpureus* also occurs in the cryosphere in high alpine areas, Antarctica [33]–[35]. In the Rwenzori Mountains, unfortunately inhabitation of *C. purpureus* around glacier had not directly observed by authors, however, *C*. *purpureus* has been detected at elevations from 2800 m to 3700 m [36] and *C. purpureus* specimen (PC0106302) taken at just below the Speke Glacier (4480 m a.s.l.) are stored in Muséum National d’Histoire Naturelle, Paris, France. These evidences would show that possibility of dispersal of spore or gemma from near glacier and deposition on the glacier surface by local wind circulation.

### Adaptation of GMGA isolate to warmer temperature

The optimum temperature of polar mosses are widly distributed from 2°C to 35°C according to species [37]. The optimum temperature for the *C*. *purpureus* isolates (25°C; Fig. 6) is normal value even in polar region, but this was higher than that for *C*. *purpureus* in Antarctica as previously reported, which was 15°C in the liquid and agar cultures [33]. Moreover, another study showed that the optimum temperature for photosynthesis in *C*. *purpureus* is around 15°C, but significant carbon fixation occurs at 5°C [35]. Although the measurement of optimum temperature by measuring fluorescence of chlorophyll used in the present study is an indirect measurement of growth (e.g., [33]), these values reflect photosynthetic ability at each temperature (e.g., [35]). Therefore, the populations of *C. purpureus* from this Ugandan glacier have likely adapted to a higher optimum temperature than Antarctic populations.

### Cold and light stress in isolated *C. purpureus*

Polar mosses tend to adapt broad range of favorable temperature. For example, relationship between net assimilation rate (NAR) and temperature of *Drepanocladus* uncinatus in Signy Island show that optimum temperature is 20°C and NAR are more than 40% from 0°C to 30°C (5°C interval) by using cold incubation sample (5°C in light/−5°C in dark) [37]. Relatively higher temperature optimum and broad range of favorable temperature is similar to our result of GMGA and *C. purpureus* isolates. Optimum temperature shift with environmental temperature change were reported from some of experiments [38], [39], but *Drepanocladus* uncinatus in Signy Island [37] did not correspond to these studies. Also in our study, optimum temperature did not shift to lower temperature inspite of preincubation temperature at 4°C. The internal temperature of the GMGAs (Fig. 5) was below the optimum (Fig. 6), therfore, the daytime internal temperature of GMGA (10°C) is not optimum but favorable for growth of Ugandan glacial *C. purpureus*. In this temperature range of internal GMGAs (−5°C to 10°C), the optimum PAR is below the warmer temperature (Fig. 6). This phenomenon may be able to explain photoinhibition from cold stress. In low-temperature conditions, PSII is inhibited due to decreased rate of repair of damaged D1 protein and increased excitation pressure [40].

Polar mosses are able to survive short-term freezing and thawing cycles in summer and prolonged freezing in winter. *Bryum argenteum* taken from tropical to polar origin showed no damage after 10 days with a temperature regime of 5°C in light/−5°C in dark, and grew slowly under these conditions [37]. In Rwenzori, *C. purpureus* live under similar dirnal cycle (from −5°C to 10°C) through year and GMGAs structure would be formed slowly. After being deposited and frozen at below −20°C for 2.5 years, regeneration of the *C*. *purpureus* from east Antarctica is very active [33]. *Ceratodon purpureus* protonemata from GMGA can be isolated and grown at 4°C after half a year of cryopreservation at −80°C. Therefore, isolate of *C. purpureus* in this study have potential to survive both short-term and prolonged freezing stress.

PAR in this natural environment is higher than optimum PAR at 5–15°C. Although we did not measure PAR directly, an automatic weather station with a radiation meter was installed beside the Stanley Plateau (N0°22′34.55′′, E 29°52′43.24′′; 4750 m above sea level) by the Stations at High Altitude for Research on the Environment project [41]. According to their data, and assuming no significant divergence with today’s conditions, the maximum diurnal seasonal shortwave radiation (c.a. 400 W/m^2^) occurs around 2∶00 PM. PAR (400–700 nm) generally comprises 50% of total solar radiation reaching the Earth’s surface. Assuming a conversion rate of 1 W/m^2^ to 4.57 µmol/m^2^/s, our estimated maximum PAR is 914 µmol/m^2^/s when maximum shortwave radiation is 400 W/m^2^. This value is higher than the optimum PAR in any temperature and this may cause photoinhibition in low temperature.

These results indicate that both low temperature and high radiation on the glacier are stress factors for *C*. *purpureus*. In acidic rivers in Japan, *Dicranella heteromalla* (Hedw.) Schimp. remains in a prolonged protonema stage for several growing seasons without producing shoots or sporophytes [42], which researchers concluded was due to the water’s extraordinarily low pH (1.9–2.1). A similar prolonged protonema phase of the moss *Scopelophila cataractae* (Mitt.) Broth was reported in copper-rich sites as well in Japan [43]. Therefore, the low temperature and high radiation stress on the glacier, may keep *C*. *purpureus* in the gemmae and protonemal stage instead of developing into shoots.

### Higher photosynthesis activity of GMGA than of isolates

The value of ETR from GMGAs was twofold that of isolates (Fig. 6), possibly due to differences in nutrient condition and the effects of other photosynthetic microorganisms. Growth conditions of moss may be more suitable in GMGA than in the artificial medium (liquid BBM) used in this study, because GMGA contains sufficient nutrients for effective growth. Yet, GMGA is not a simple aggregation of only moss, but also contains many other microorganisms. For example, we observed *Cylindrocystis brebissonii* cells and red snow algae [44], which is related to the green algae commonly found in supraglacial environments, in the GMGAs. Both moss and green algae affect the total photosynthetic activity of GMGAs.

### Possible process of GMGA formation

If biological material in sites without GMGAs form a thick deposition layer (more than a few millimeters), the temperature below the surface would increase to above 0°C, the same as in GMGAs. If this is so, then the invasion of gemmae of *C. purpureus* adapted to warmer temperatures on the cold glacier surface can be attributed to this increased subsurface temperature. Similar temperature increases occur in other glaciers (e.g., Qiyi Glacier); however, GMGA-like structures and growth of moss gemmae have not been found on any other glacier, despite studies of glacier biology being conducted around the world [45]. This may relate to possible inhabitation of *C. purpureus* near the glacier and unique features of the Rwenzori; namely, the lack of a clear seasonal temperature cycle. On Stanley Plateau, diurnal temperature change in all seasons is in a range of approximately 0°C to 5°C [41]. Consequently, long periods of freezing do not exist, permitting microorganisms to grow throughout the year. Therefore, we suppose that at least these two factors (the internal temperature rise and the long growth season) may contribute to the formation of GMGAs.

However, GMGAs are disproportionally dominant near the glacier terminus. The high number of GMGAs observed at ST1 must be supported by factors specific to that site. Although our data does not let us reach firm conclusions about the distribution of GMGAs, downward transpotation by surface melting water and availability of sunlight is a likely candidate factor.

Glacier surface melt of Stanley Plateau homogenously spread over the ice area and remarkable water channels on surface are few. Takeuchi [46] speculated cryoconite granules (around 1 mm diameter) are more stable from meltwater than unicellular microorganisms due to larger size character. GMGAs are much larger diameter than typical cryoconite (Fig. 3) seems to be more stable on the ice. Also GMGAs penetrate few mm into ice due to radiation warming (Fig. 2b). These also prevent to wash this material to downward. Therefore, downward transportation of well-developed GMGAs seems unlikely happened.

During the biological growth season, supraglacial light conditions generally change based on depth of snow cover. In the early melt season seasonal snow is removed by melting to expose the glacial ice surface to sunlight at lower elevations only. The snow line then retreats until reaching equilibrium line altitudes at the end of melt season. This leaves the entire surface of the ablation zone snow-free, making it an available habitat for photosynthetic microorganisms. As a result, the biodiversity of glacier microbial communities changes with elevation [44], [46]–[48].

In early February 2013, the entire glacier surface was covered by snow except for a steep slope at the glacier terminus, where ST1 is located. The snow cover near ST2 was 0.85 m deep, but near ST1, zero or a few centimeters of snow cover were observed. Because snow blocks radiation, these differences in snow depth cause variable light conditions and GMGA internal temperatures. Although the precise factors causing this difference in snow depth are unknown, slope angle and wind erosion likely to cause gradients in snow cover. We observed similar types of steep slopes on all edges of this glacier and other glaciers located beyond the ridge (Margarita Glacier: Fig. 2a); however, further observations and measurements must be necessary.

### Direct ecological linkage between glacier and glacier foreland

In the glacier foreland immediately adjacent to the glacier terminus, we found an abundance of dried GMGAs on rock surfaces, which were likely left on the freshly bared subglacial rocks after glacier retreat. The temperature of these dried GMGAs on the rocks reaches approximately 20°C and conditions appear much drier than on the glacier, where water is supplied by melting (Fig. 5). Dried GMGAs create a soil-like structure on the abiotic rock surface with gametophyte of different dominant Bryophyta (*Bryum* sp.). The succession of dominant bryophyte species from *C*. *purpureus* gemmae to *Bryum* sp. shows that GMGAs had changed after leaving the glacier. Previous studies conceptually proposed the linkage between glacier and glacier foreland by nutrient connection [49] and outwash of cryoconite granules [2]. Otherwise, our findings indicate that GMGAs accumulate as a soil-like structure on the abiotic rock surface, directly linking the glacier and glacier foreland ecosystems.

Furthermore, recently another linkage between glacier and glacier foreland was found from subglacial environment [50], [51]. In Canadian Arctic, varaeties of mosses regenerate from old populations, which had been entombed in subglacial environment from Little Ice Age (LIA) and recently released onto ice –free glacier foreland due to glacial retreat [50]. These evidences show releases of developed biological material from both supraglacier and subglacier supply the stable ecological substructure to glacier foreland ecology.

If the glacier disappears due to climate change and/or albedo reduction, this unique glacial ecosystem and its contribution to the glacier foreland will also disappear. Many other tropical glaciers that are expected to disappear in the near feature [52], [53], which may also contain unique biota that are under threat. In this respect, the tropical glacial ecosystem is an urgent subject of study to understand the biodiversity.
