# Correction: Novel Biogenic Aggregation of Moss Gemmae on a Disappearing African Glacier

**DOI:** 10.1371/journal.pone.0123375

**Published:** 2015-04-01

**Authors:** The PLOS ONE Staff

The affiliations for the sixth and seventh authors are incorrect. Hideaki Motoyama and Satoshi Imura are not affiliated with #3 but with #2: National Institute of Polar Research, Tachikawa, Tokyo, Japan.
